# Data relating to carbonaceous components in Songkhla Lake sediments, Thailand

**DOI:** 10.1016/j.dib.2019.01.039

**Published:** 2019-01-19

**Authors:** Siwatt Pongpiachan, Qiyuan Wang, Li Xing, Guohui Li, Yongming Han, Junji Cao

**Affiliations:** a NIDA Center for Research & Development of Disaster Prevention & Management, School of Social and Environmental Development, National Institute of Development Administration (NIDA), 118 Moo 3, Sereethai Road, Klong-Chan, Bangkapi, Bangkok 10240, Thailand; b SKLLQG and Key Lab of Aerosol Chemistry & Physics, Institute of Earth Environment, Chinese Academy of Sciences (IEECAS), Xi’an 710061, China

**Keywords:** Organic carbon (OC), Elemental carbon (EC), Sediment cores, Songkhla lake

## Abstract

The focus of this research was to present a data article associated with organic carbon (OC) and elemental carbon (EC) preserved in lake sediments. Descriptive statistics were applied in this dataset. Sediment cores were sliced immediately at the following layers: 0–20; 20–40; 40–60; 60–80; 80–100; 100–120; 120–140; 140–160; 160–180; 180–200; 200–220; 220–240; 240–260; 260–280; 280–300; 300–320; 320–340; 340–360; 360–380; 380–400; 440–460; 460–480; 480–500; 500–520; 520–540; 540–560 and 560–580 mm of depth. Chemical analysis of OC (i.e. OC1, OC2, OC3, OC4), EC (i.e. EC1, EC2, EC3), and the pyrolyzed organic carbon (OP) (i.e. OP1, OP2, OP3, OP4, OP5, OP6, Char, Soot) contents was conducted by using a DRI Model 2001 Thermal/Optical Carbon Analyzer. The chemical characterization coupled with statistical analysis reveal that vehicle exhausts are the most prominent factor governing OC/EC data detected in core sediments. These data underline some noticeable concerns associated with ecotoxicology and environmental safety of residents surrounding the Songkhla Lake.

**Specifications table**

**Table:** 

Subject area	Environmental Sciences
More specific subject area	Environmental Chemistry
Type of data	Table, text file, graph, figure
How data was acquired	Entire sediment samples were quantitatively identified employing a DRI Model 2001 Thermal/Optical Carbon Analyser (Desert Research Institute, Division of Atmospheric Sciences 2215 Raggio Parkway Reno, NV 89506) [1], [2]
Data format	Raw data, analysed.
Experimental factors	A gravity corer (i.e. a PVC plastic tube 12 cm in diameter 1.2 m in length) was specifically designed for this study. All materials used for core sectioning were washed carefully with detergent and water, and rinsed successively with methanol and dichloromethane prior to removing the frozen core from the freezer. The freeze dried lake sediment samples were ground and homogenized with an agate mortar and sieved though a 200-mesh sieve. The sample pre-treatment procedure has been clearly described in previous studies [3], [4].
Experimental features	OC/EC contents analyzed by a DRI Model 2001 Thermal/Optical Carbon Analyzer.
Data source locations	The Thale Noi Lake (TNL) is located at 7° 46′ 00″ N 100° 09′ 11″ E, which is the largest lagoon lake in Thailand. Three undisturbed sediment cores were collected from the northern, central, and southern parts of the TNL in August 2017 when the water level ranged between 150 and 170 cm.
Data accessibility	Data available within article.
Related research article	Pongpiachan, S., Tipmanee, D., Choochuay, C., Hattayanone, M., Deelaman, W., Iadtem, N., Bunsomboonsakul, S., Palakun, J., Poshyachinda, S., Leckngam, A., Somboonpon, P., Panyaphirawat, T., Aukkaravittayapun, S., Wang, Q., Xing, L., Li, G., Han, Y., and Cao, J., 2019. Vertical profile of organic and elemental carbon in sediments of Songkhla Lake, Thailand. *Limnology* (In Press) (https://doi.org/10.1007/s10201-018-0568-9)

**Value of the data**

• Analytical data can be used as a base-line data for OC/EC concentration levels in sediments of Songkhla Lake.
• OC/EC data plays a crucial role in governing climate system, therefore, continuous chemical characterization of carbonaceous compositions in lake sediments is undoubtedly essential for atmospheric modellers to reconstruct the paleoclimate.
• Data displayed here can be served as benchmarks for other studies highlighting in the field of ecotoxicology to evaluate sediment-water partitioning of POPs by applying OC/EC contents.
• Data of OC/EC ratios can be applied to assess the impacts of vehicle exhausts, biomass burnings and volcanic eruptions. This was about giving policy makers the actual tools that will enable them to develop pollution control policy that can also be referred to as scientific evidence-based decision-making.

## 1 Data

Table 1 displays vertical profile of OC1, OC2, OC3, and OC4 in Songkhla Lake sediments. Table 2 shows concentrations of EC1, EC2, and EC3 in different sediment layers. Table 3 illustrates contents of OP1, OP2, OP3, OP4, OP5, and OP6 in Songkhla Lake sediments.

**Table 1:** Vertical profile of OC1, OC2, OC3, and OC4 in Songkhla Lake sediments.

**Sample ID**	**Depth [mm]**	**OC1 [mg g**^**−1**^**]**	**OC2 [mg g**^**−1**^**]**	**OC3 [mg g**^**−1**^**]**	**OC4 [mg g**^**−1**^**]**
SL101	20	0.45	30.3	161	13.4
SL102	40	0.57	34.9	173	14.6
SL103	60	0.56	31.7	164	15.6
SL104	80	0.53	24.9	147	13.5
SL105	100	0.21	18.1	121	9.90
SL106	120	0.85	34.1	177	18.2
SL107	140	0.54	53.4	216	24.7
SL108	160	1.09	94.2	268	33.5
SL109	180	1.94	135	306	42.9
SL110	200	0.79	77.6	246	28.0
SL111	220	0.43	51.5	221	19.0
SL112	240	0.47	31.8	177	16.0
SL113	260	0.88	99.0	277	36.8
SL114	280	0.97	92.2	268	36.5
SL115	300	1.00	102	265	31.2
SL116	320	1.66	110	187	21.5
SL117	340	0.34	17.6	80.3	5.30
SL118	360	0.96	54.6	151	14.8
SL119	380	3.19	138	203	24.8
SL120	400	2.58	122	194	22.6
SL122	440	2.94	156	216	27.8
SL124	480	3.31	116	182	23.4
SL125	500	4.00	211	218	50.1
SL126	520	2.65	170	207	50.9
SL127	540	2.30	149	194	46.8
SL128	560	1.55	61.9	161	22.2
SL129	580	1.40	79.3	164	26.1

**Table 2:** Vertical profile of EC1, EC2, and EC3 in Songkhla Lake sediments.

**Sample ID**	**Depth [mm]**	**EC1 [mg g**^**−1**^**]**	**EC2 [mg g**^**−1**^**]**	**EC3 [mg g**^**−1**^**]**
SL101	20	211	6.73	0.39
SL102	40	241	6.12	0.39
SL103	60	235	6.18	0.51
SL104	80	220	5.74	0.38
SL105	100	187	4.49	0.26
SL106	120	321	7.35	0.45
SL107	140	378	10.8	0.44
SL108	160	464	11.6	0.75
SL109	180	555	16.1	0.67
SL110	200	392	10.1	0.60
SL111	220	256	4.75	0.47
SL112	240	172	3.71	0.50
SL113	260	469	22.7	0.57
SL114	280	495	20.9	0.61
SL115	300	445	9.79	0.64
SL116	320	339	1.64	0.48
SL117	340	72.9	1.25	0.25
SL118	360	191	1.37	0.46
SL119	380	417	2.80	0.88
SL120	400	376	2.69	0.80
SL122	440	477	3.08	0.43
SL124	480	383	2.19	0.49
SL125	500	642	9.21	0.54
SL126	520	622	5.47	0.44
SL127	540	491	3.09	0.38
SL128	560	399	2.95	0.29
SL129	580	328	3.00	0.42

**Table 3:** Vertical profile of OP1, OP2, OP3, OP4, OP5, and OP6 in Songkhla Lake sediments.

**Sample ID**	**Depth [mm]**	**OP1 [mg g**^**−1**^**]**	**OP2 [mg g**^**−1**^**]**	**OP3 [mg g**^**−1**^**]**	**OP4 [mg g**^**−1**^**]**	**OP5 [mg g**^**−1**^**]**	**OP6 [mg g**^**−1**^**]**	**Char [mg g**^**−1**^**]**	**Soot [mg g**^**−1**^**]**
SL101	20	25.5	39.2	67.8	198	204	206	0.77	1.12
SL102	40	22.4	48.8	79.4	213	231	235	1.01	1.03
SL103	60	25.7	50.3	95.6	215	224	229	0.97	1.07
SL104	80	19.0	45.8	85.5	202	211	215	0.75	0.92
SL105	100	47.7	73.7	101	179	182	184	0.53	0.75
SL106	120	77.3	122	182	N.D.	260	300	3.03	1.16
SL107	140	62.1	91.8	156	N.D.	N.D.	352	4.12	1.77
SL108	160	91.1	127	204	N.D.	405	440	3.64	1.90
SL109	180	109	150	221	281	520	533	3.40	2.55
SL110	200	77.3	96.8	127	375	381	383	1.39	1.69
SL111	220	62.7	81.2	105	253	254	254	0.26	0.81
SL112	240	57.5	77.4	103	171	171	172	0.05	0.67
SL113	260	81.6	102	124	432	446	454	2.25	3.52
SL114	280	66.3	91.2	119	N.D.	173	495	N.D.	3.29
SL115	300	74.4	99.3	128	N.D.	410	425	3.30	1.68
SL116	320	58.1	76.8	105	310	315	320	2.93	0.34
SL117	340	30.1	39.0	46.8	70.4	70.7	71.1	0.29	0.25
SL118	360	56.4	66.7	96.8	182	183	184	1.09	0.28
SL119	380	45.1	73.1	100	349	360	369	7.75	0.60
SL120	400	54.8	70.4	98.8	320	328	332	6.98	0.55
SL122	440	61.5	77.1	95.9	395	408	417	9.73	0.57
SL124	480	44.9	72.8	113	213	352	360	3.74	0.43
SL125	500	78.2	96.8	270	N.D.	96.8	322	51.8	1.58
SL126	520	93.2	180	380	N.D.	24.6	555	9.84	0.88
SL127	540	41.3	75.4	203	N.D.	12.2	477	2.28	0.57
SL128	560	63.0	155	232	N.D.	167	355	7.30	0.54
SL129	580	20.9	33.2	105	N.D.	27.0	325	0.50	0.56

## 2 Experimental design, materials and methods

### Dataset area

**Fig. 1:**
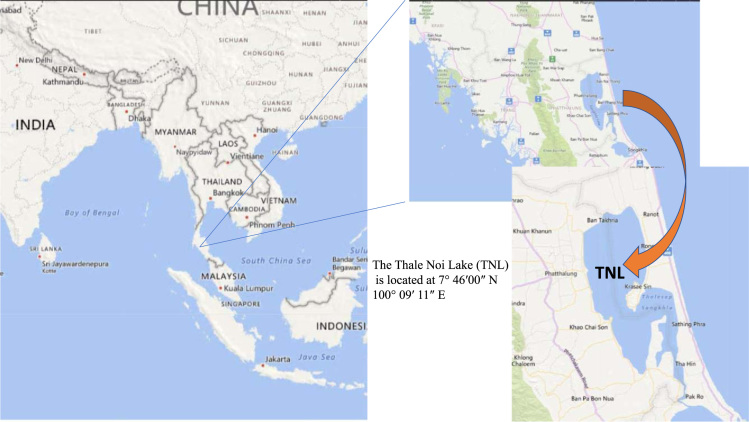
Map of the sampling site location at TNL, Songkhla Lake, Thailand.

All sediment samples were collected at the Thale Noi Lake (TNL), which is the second largest lagoon lake in Southeast Asia. TNL became internationally regarded as an ecosystem dynamic hotspot in 1975 when the Ministry of National Resources and Environment (MNRE) and in conjunction with the International Union for Conservation of Nature (IUCN) declared it a Protected Area Category III (Natural Monuments). TNL can be further separated into four subareas namely Melaleuca forests (170 km^2^), Rice Paddies (153 km^2^), Swamp (109 km^2^), and Open Water (28 km^2^). It is also crucial to underline that TNL is located in the northern part of Thale Luang, Thale Sap, and Songkhla Lake. The area around TNL constructs of farmland, forests, and swamps. There is no main river flowing through this area, but sediment loads from many small man-made canals as well as run-off water from the high steep mountains is observed [5]. The sediment core samples of TNL were collected from three sites (Fig. 1).

### Sample collection and analytical procedures

A sediment core sampling equipment (i.e. a transparent PVC plastic tube 12 cm in diameter 1.2 m in length) was carefully dropped from a vessel. All sampling equipment employed for sediment sampling were precleaned cautiously with detergent and deionized water, and rinsed continuously with methanol and dichloromethane. Further information related with QA/QC protocols were strictly followed the standard operating procedure for the USGS Reston, Virginia Environmental Organic Geochemistry Laboratory Appendix 3 (https://water.usgs.gov/nrp/biogeochemical-processes-in-groundwater/forms/SOP_LMWOA_05272015_FINAL_Website.pdf). Pre-treatment processes of freeze-dried core sediment samples were clearly mentioned in earlier investigations and will not be discussed here [3], [4]. All core sediment samples were both qualitatively and quantitatively characterized by using a DRI Model 2001 Thermal/Optical Carbon Analyser (Desert Research Institute, Division of Atmospheric Sciences 2215 Raggio Parkway Reno, NV 89506) [1], [2]. The application of an analytical equipment is fundamentally relied on the advantageous oxidation of OC and EC components at numerous heating conditions. Its function relies on the fact that organic compounds can be volatilised from the sample deposit in a non-oxidising He atmosphere while EC must be combusted by an oxidiser. According to the IMPROVE_A method, OC1, OC2, OC3, and OC4 represent the quantities of carbon evolved from the filter during each of four non-oxidizing heat ramps at 120 °C, 250 °C, 450 °C, and 550 °C, respectively. Four different OC fractions (i.e. OC1, OC2, OC3, and OC4) were carefully selected because of its useful parts of the source fingerprints [1], [6], [7], [8], [9]. Then a 2% O_2_/98% He was introduced, and the oven temperature was raised to 550 °C, 700 °C and 800 °C, producing three EC fractions: EC1, EC2, and EC3. Total carbon (TC, no carbonate carbon included in this study) is the sum of all carbon fractions. Evolved carbon gases were oxidized to CO_2_, then reduced to CH_4_ for detection with a flame ionization detector. Some of the organic carbon chars in the inert He environment, as indicated by decreased reflectance or transmittance of the laser from the sample deposit. Once in the oxidizing atmosphere, the pyrolyzed organic carbon (OP) leaves the filter. The quantity of OP is defined as the carbon that evolves to the time at which the laser reflectance or transmittance achieves its initial value. OP1, OP2, and OP3 are reflectance-corrected OP, corresponding the minimum (the beginning of the laser reflectance that achieves its initial value), middle (the stable condition of the laser reflectance that achieves its initial value), and maximum OP (the last point that the laser reflectance start to leave its initial value). Correspondingly, OP4, OP5, and OP6 indicate the transmittance-corrected minimum, middle, and maximum OP. For more details of analytical processes of OC/EC were previously mention in other studies and will not be discussed here [1], [2].
